# Dermoscopic Features as Predictors of *BRAF* Mutational Status and Sentinel Lymph Node Positivity in Primary Cutaneous Melanoma

**DOI:** 10.5826/dpc.1102a40

**Published:** 2021-04-12

**Authors:** Nika Filipović, Mirna Šitum, Marija Buljan

**Affiliations:** 1 Department of Dermatology and Venereology, University Hospital Centre Sestre Milosrdnice, Zagreb, Croatia; 2 Department of Dermatovenereology, School of Dental Medicine, University of Zagreb, Croatia

**Keywords:** melanoma, *BRAF*, sentinel lymph node, dermoscopy

## Abstract

Dermoscopy is a diagnostic tool widely used in clinical practice for the detection of skin tumors, especially early stages of melanoma. Recent studies have shown that different dermoscopic features are associated with important prognostic parameters of melanoma, such as BRAF mutational status and sentinel lymph node status. More than half of all melanomas harbor a mutation in the BRAF oncogene. The current management of advanced-stage melanomas is greatly determined by the presence or absence of a mutation in this gene, as targeted therapy with *BRAF* kinase inhibitors is one of the first therapeutic choices for these patients. Sentinel lymph node status is one of the most significant predictors of a melanoma patient’s survival. Recent studies have shown that different dermoscopic patterns are also associated with sentinel lymph node status. This short article reviews studies that investigated correlations between dermoscopic features, *BRAF* mutation status and sentinel lymph node status.

## Introduction

Melanoma is one of the most aggressive malignant skin tumors, with a rapidly increasing incidence over the course of the past 50 years worldwide, especially in fair-skinned Caucasian populations [1]. In 2018, approximately 300,000 new melanoma cases were registered globally [2].

Since the identification of *BRAF* as an important oncogene in melanoma in 2002 [3], new therapeutic options have been developed and successfully implemented. The *BRAF* gene encodes a serine-threonine kinase that is a member of the MAPK [mitogen-activated protein kinase] signaling pathway. Approximately 50%–60% of melanomas harbor a *BRAF* gene mutation, with the most common oncogenic alteration involving codon 600 [3]. A growing body of literature has demonstrated that different patterns of oncogene mutations correlate with different histological and clinical features of melanoma. In particular, there is a higher frequency of *BRAF* mutations in melanomas of younger patients, melanomas located on the trunk, lesions of the superficial spreading histological subtype, and melanomas that develop on skin without chronic actinic damage [4–9].

Combined targeted therapy with small-molecule inhibitors of mutant *BRAF* and down-stream kinase MEK (MAPK inhibitors), as well as immunotherapy with inhibitors of PD-1 (programmed cell death receptor 1), represent today’s first therapeutical choices for the majority of patients with metastatic melanoma [10]. Since this approach has significantly improved overall survival, assessment of *BRAF* mutational status in tumor tissue, with standardized molecular methods, is crucial for treatment decisions.

Furthermore, it has been demonstrated that melanomas harboring *BRAF* mutations share certain morphological features detectable with noninvasive diagnostic tools such as dermoscopy [11]. In the past decades, dermoscopy has become a method widely used in clinical practice for detecting skin tumors, especially early stages of melanoma. Since *BRAF*-mutated melanomas show specific histomorphological features, specific dermoscopic features could be anticipated as well. However, only a few studies with heterogeneous results have been published on the relationship between dermoscopic patterns of melanoma and *BRAF* mutational status. As previously mentioned, since the current management of advanced-stage melanomas is greatly determined by the presence or absence of *BRAF* mutations, identifying specific dermoscopic features associated with *BRAF* mutational status before tumor excision could be of great importance in making further diagnostic and therapeutic decisions.

Sentinel lymph node (SLN) biopsy is a diagnostic procedure used to detect occult regional node melanoma metastases. According to international consensus and the latest American Joint Committee on Cancer classification from 2018 [12], SLN biopsy is generally indicated in melanomas with a thickness of 0.8 mm or more, and in lesions with ulceration. So far only sporadic studies regarding the correlation between dermoscopic patterns and SLN status have been conducted. Therefore, identifying specific dermoscopic features associated with SLN positivity could also be of great significance to clinicians in making a diagnostic-therapeutic algorithm for melanoma patients.

## Dermoscopic Features and *BRAF* Mutational Status

A study by Pozzobon et al [13] was one of the first to investigate the correlation between dermoscopic features and MAPK mutational status. That study identified a significant association between dermoscopic regression, designated as “peppering”, and *BRAF* mutations (OR = 1.68; 95% CI, 1.089–2.581, P = .015). In addition, after acral and facial melanomas [which may show different dermoscopic patterns [14,15] were excluded from analysis, the presence of dermoscopic ulceration was also associated with *BRAF* mutation status (OR = 2.64; 95% CI, 1.032–6.754; P = .032).

Bombonato et al [16] reported that dermoscopic ulceration and irregular peripheral streaks are positive predictors of *BRAF*-mutated melanoma. It is well known that the dermoscopic presence of streaks is a sign of tumor growth and proliferation; in fact, streaks correspond to the presence of peripheral nests of tumor cells. However, they can also be seen in nevi (eg, Spitz/Reed nevi). On the other hand, the same study showed that the dermoscopic presence of dotted vessels was a negative predictor of *BRAF*-mutated melanomas. Only 10% of lesions with dotted vessels in that study were *BRAF*-mutated melanomas (P = .004). In contrast to the study by Pozzobon et al [13], regression in the form of dermoscopic peppering did not correlate with *BRAF*-mutated melanomas [16].

Fargnoli et al [17] did not identify any significant differences between dermoscopic features of *BRAF*-mutated and wild-type melanomas. These authors suggested that the limited number of dermoscopic images was the main limitation of their study.

Armengot-Carbó et al [18] showed a strong association between the presence of blue-white veil in dermoscopy and *BRAF* mutations (P = .003). The blue-white veil corresponds to a large nest of intensely pigmented tumor cells located under a thickened epidermis [19–21]. Accordingly, histomorphological studies revealed that *BRAF*-mutated melanomas had a thicker epidermis and more pigmented cells with a greater tendency to form nests than wild-type melanomas [22,23]. Unfortunately, the study by Bombonato et al did not report data regarding this important dermoscopic pattern [16].

Furthermore, the study by Armengot-Carbó et al [18] did not show correlation between dermoscopic ulceration, dotted vessels and *BRAF* mutational status, as observed before [16]. This could be explained by the fact that, in the study by Bombonato et al [16], genetic testing was performed mainly when there was a clinical indication, that is, in predominantly thick melanomas. Consequently, there was also a higher mean Breslow thickness and higher ulceration frequency, and consequently a higher frequency of dermoscopic ulceration in their study, while in a study by Armengot-Carbó et al [18] there were no significant differences in Breslow thickness or histological ulceration. Although the presence of vascularization is in general a sign of tumor invasion and progression, dotted vessels are predominantly found in thin melanomas [24]. This could have affected their results due to the predominance of thicker melanomas in the *BRAF*-mutated group [16]. In addition, it should be noted that the number of melanomas with dermoscopic ulceration was higher than those with histological ulceration [13,16,25], probably due to the higher sensitivity of dermoscopy, which can detect small peripheral ulcerations not found by histopathology due to sampling techniques [18].

Recently, Gouillon et al [26] reported an observational study of more than 100 melanomas that were compared dermoscopically and genetically. Pseudopods and radial projections were both observed more frequently in *BRAF*-mutated melanomas. Since these structures can be combined into “irregular peripheral streaks,” these results are concordant with findings reported by Bombonato et al [16] and Armengot-Carbó et al [18]. Blue-gray peppering and white scar-like areas, dermoscopic features related to histological regression [27], were also more frequently found in *BRAF*-mutated melanomas than in wild-type lesions (P = .044). Concordant with the results of Armengot-Carbó et al [18], blue-white veil was more frequently present in the *BRAF*-mutated group (P = .007). Additionally, Gouillon et al reported for the first time ever the parallel-ridge pattern as a negative predictor of *BRAF* mutational status in acral lentiginous melanomas, as it was more frequently found in the wild-type melanoma group (P = .022) [26].

## Dermoscopic Features and Sentinel Lymph Node Positivity

As metastases from melanoma most frequently develop in lymph nodes, SLN biopsy has emerged as a key diagnostic tool for determining whether cancer has spread to the regional lymph nodes. This minimally invasive procedure has successfully replaced elective lymph node dissection in the management of melanoma patients [28,29]. According to several studies, histological features of the primary lesion and SLN biopsy are the most significant predictors of a melanoma patient’s survival [30,31]. González-Álvarez et al [32] investigated the association between dermoscopic structures and SLN status, and found that the presence of an atypical pigmented network was associated with a negative SLN. A dermoscopic pigmented network represents pigmented rete ridges histologically, so thicker melanomas lose these rete ridges due to tumor progression and invasion of the dermis. Consequently, it is evident that in thick melanomas, where SLN biopsy is done, a dermoscopic atypical pigmented network is often not found. On the other hand, the presence of dermoscopic ulceration and blotches [area of homogeneous dark pigmentation] correlated with a positive SLN. The presence of a blue-white veil, atypical vessels and regression structures was not significantly correlated to SLN status.

Pagnanelli et al [33] failed to identify any predictive dermoscopic criteria for SLN positivity in melanomas thicker than 1 mm. This outcome could be explained by the fact that only 23% of patients studied had melanomas thicker than 1 mm requiring SLN biopsy.

## Conclusions

Even though dermoscopy cannot replace molecular methods and histopathology in determining *BRAF* mutational status and SLN status, it could be a useful additional diagnostic tool in predicting these melanoma features. Different dermoscopic patterns (eg, blue-white veil, ulceration, peppering) have been identified as significant predictors of *BRAF* mutational status and SLN status, and therefore could be of great significance in making diagnostic-therapeutic algorithms for melanoma patients. However, further studies are needed to investigate these findings and identify other dermoscopic criteria associated with *BRAF* mutations and SLN positivity.
